# Whole genome analysis reveals aneuploidies in early pregnancy loss in the horse

**DOI:** 10.1038/s41598-020-69967-z

**Published:** 2020-08-07

**Authors:** Charlotte A. Shilton, Anne Kahler, Brian W. Davis, James R. Crabtree, James Crowhurst, Andrew J. McGladdery, D. Claire Wathes, Terje Raudsepp, Amanda M. de Mestre

**Affiliations:** 1 Department of Comparative Biomedical Sciences, The Royal Veterinary College, University of London, London, UK; 2 Department of Veterinary Integrative Biosciences, Texas A&M University, College Station, TX USA; 3 Equine Reproductive Services (UK) Ltd., North Yorkshire, UK; 4 Newmarket Equine Hospital, Newmarket, Suffolk UK; 5 Rossdales Equine Practice, Newmarket, UK; 6 Department of Production and Population Health, The Royal Veterinary College, University of London, Hatfield, UK

**Keywords:** Developmental biology, Genetics

## Abstract

The first 8 weeks of pregnancy is a critical time, with the majority of pregnancy losses occurring during this period. Abnormal chromosome number (aneuploidy) is a common finding in human miscarriage, yet is rarely reported in domestic animals. Equine early pregnancy loss (EPL) has no diagnosis in over 80% of cases. The aim of this study was to characterise aneuploidies associated with equine EPL. Genomic DNA from clinical cases of spontaneous miscarriage (EPLs; 14–65 days of gestation) and healthy control placentae (various gestational ages) were assessed using a high density genotyping array. Aneuploidy was detected in 12/55 EPLs (21.8%), and 0/15 healthy control placentae. Whole genome sequencing (30X) and digital droplet PCR (ddPCR) validated results. The majority of these aneuploidies have never been reported in live born equines, supporting their embryonic/fetal lethality. Aneuploidies were detected in both placental and fetal compartments. Rodents are currently used to study how maternal ageing impacts aneuploidy risk, however the differences in reproductive biology is a limitation of this model. We present the first evidence of aneuploidy in naturally occurring equine EPLs at a similar rate to human miscarriage. We therefore suggest the horse as an alternative to rodent models to study mechanisms resulting in aneuploid pregnancies.

## Introduction

Early pregnancy loss (EPL) represents the largest contributor to reproductive failure in many mammalian species^1–3^. In women EPL causes emotional distress, and in domestic species breeding economics and welfare is negatively impacted. Despite intensive management strategies, between 5 and 10% of confirmed equine (*Equus caballus*) pregnancies end in the first 8 weeks^2^. This incidence has remained relatively stable over the past few decades^2,4^. While many factors increase a mare’s risk of EPL^5^, these are not always causative per se. Over 80% of equine EPLs receive no formal diagnosis^2^, with the remaining 20% being diagnosed as either infectious (~ 4%) or non-infectious (~ 16%) in nature^2,6^. In unexplained equine EPLs, genetic abnormalities are often attributed as a potential underlying cause, yet this remains unproven beyond a few case studies detailing balanced autosomal translocations in otherwise phenotypically normal individuals diagnosed with infertility^7–12^. Advancing maternal age has been consistently associated with pregnancy loss in many species^5,13^. Whilst an abnormal uterine environment is likely to be a contributing factor, experimental transfer of embryos from young mares into old mares (and vice versa) have suggested that this is not the main age-related underlying cause of EPLs^14^. Oocytes from aged mares are of lower quality compared with younger fertile mares^15,16^, indicating oocyte quality as a more likely risk factor for pregnancy loss.

Aneuploidy (gain or loss of a whole chromosome) is well documented in human spontaneous abortion^17^ and is associated with advancing maternal age. Some human autosomal trisomies (13, 18, and 21) have been identified in infants following live births, although these usually present with significant developmental pathologies^18^ with only trisomy 21 individuals surviving to adulthood^19^, and no documented surviving monosomies. The remainder of chromosomes that experience autosomal aneuploidy have only been identified in spontaneous abortion samples, leading to the theory that certain chromosomes do not tolerate aneuploidy and are therefore embryonic/fetal lethal. While more commonly reported in humans, aneuploidy has been noted occasionally in individual cases in domesticated species including live born calves^20^ and foals^21–27^. Equines reported to survive to term are often euthanised at a young age due to extreme developmental defects^21–27^. In species with reported live born individuals with autosomal aneuploidies, all are trisomies with no reported monosomies in the literature^28^. This indicates that while some trisomies may be tolerated to term, monosomies are always lethal at some stage during pregnancy. Very few studies have investigated aneuploidy rates in spontaneous abortion of non-human species. A single study into bovine spontaneous abortions^29^ identified four trisomies in 55 individuals. Investigations analysing in vitro or in vivo generated blastocysts have identified chromosomal abnormalities in a number of species including equine^30^, bovine^31^, and ovine^32^. It is important to note that these blastocysts have been purposely prevented from achieving a pregnancy, and therefore the outcome of these chromosomal abnormalities cannot be determined. The relevance of these observations in blastocysts to naturally conceived pregnancies is untested. Previous attempts to identify chromosomal abnormalities in equine abortions have been largely unsuccessful^33,34^, but successful isolation of conceptus material from EPLs^35^ opens up the possibility for investigation into genetic and chromosomal causes of EPL in the mare. While karyotyping has been used as the gold standard for aneuploidy analysis, these assays are time consuming and require specialised experience to generate results in large numbers. New methods are emerging that may replace karyotyping as gold standard, allowing for higher throughput analysis and diagnosis of aneuploidy, even in degraded conceptus tissues. Whole genome sequencing (WGS) is one potential method, however the cost is still too high for this to be a viable solution. Single Nucleotide Polymorphism (SNP) Arrays are a lower cost, high throughput method that may offer an answer. A low-density SNP array (EquineSNP50 Genotyping BeadChip, Illumina) has previously been used to detect aneuploidy in two live born horses suspected of chromosomal abnormalities^23^, offering evidence for the validity of this methodology. More recently, a higher density array featuring 670 k SNPs has become available^36^ (Axiom) allowing simultaneous detection of large genetic variants such as aneuploidy, and smaller structural variants.

In order to explore whether aneuploidy is a feature of failed pregnancies in domesticated animal species, we utilised methods previously reported^35^ to generate a large bank of conceptuses from naturally occurring clinical cases of EPL in mares. We hypothesised that due to the rarity of aneuploidy in foals born at term, the majority of aneuploidy presents as embryonic/fetal lethal. It will be detectable in both placental and fetal compartments of EPL conceptuses consistent with possible origins in maternal meiosis. We hypothesise that aneuploidy will be rare in phenotypically normal pregnancies and adult horses. The primary aim of this study was to quantify the frequency and characteristics of aneuploidy associated with EPL in the mare.

## Results

### Descriptive data of SNP array sample population

**Figure 1:**
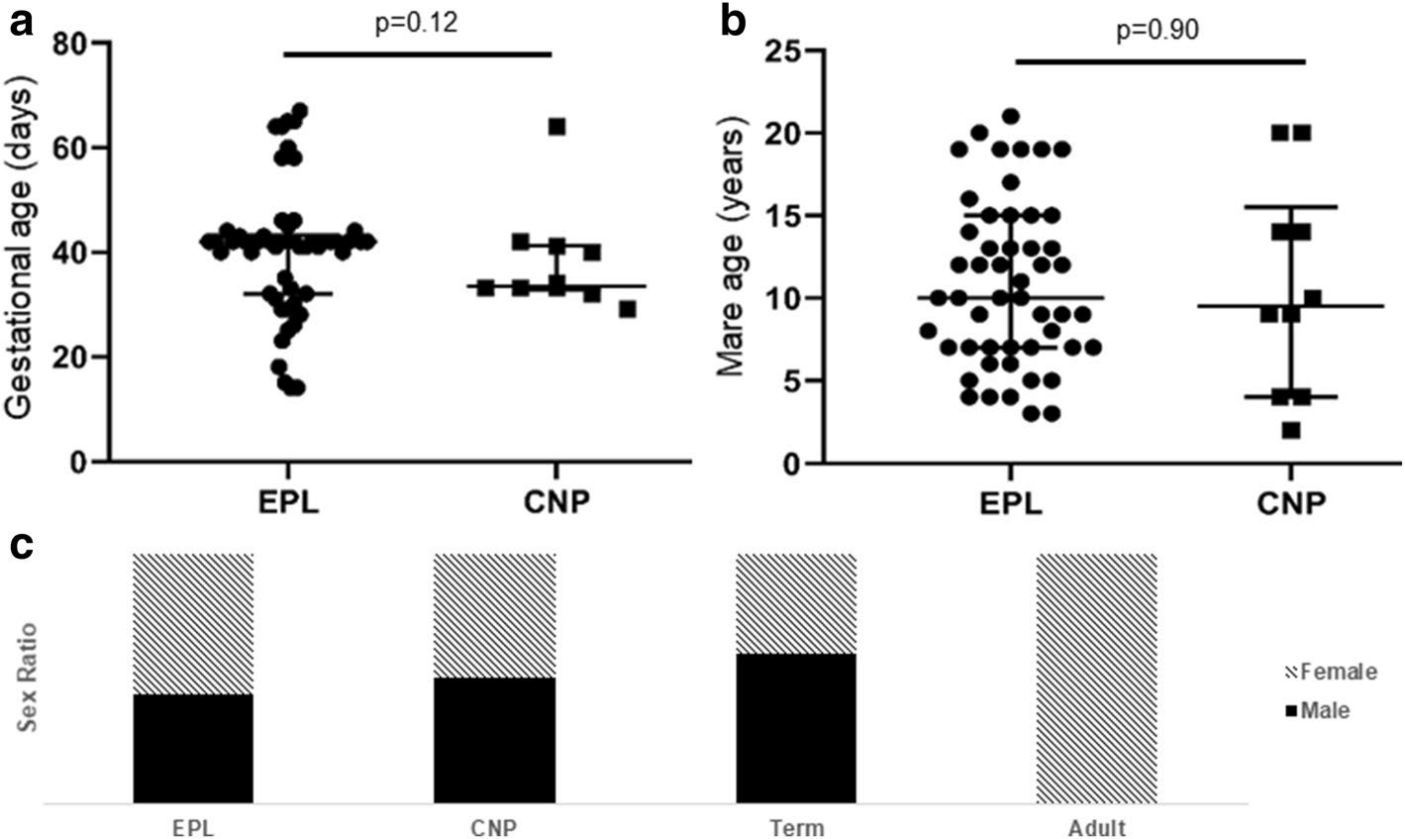
Description of sample population on the SNP array. No significant difference was found in the average (**a**) gestational age or (**b**) mare age of pregnancies within the early pregnancy loss (EPL) and clinically normal pregnancy (CNP) groups. Mean with standard deviation plotted. (**c**) Males and females were equally represented on the array for the EPL, CNP, and healthy term placentae. All adults were females.

The median gestational age of the early pregnancy loss (EPL; n = 55) and clinically normal pregnancies (CNP; n = 10) conceptuses was 42(± 12.5SD) and 33.5(± 10.1) days respectively (Fig. 1a). Chorioallantois tissue was obtained from clinically normal term births (n = 5) following the delivery of a healthy foal. The range of gestational ages was 14–67 and 29–64 days for EPL and CNP respectively and were not significantly different (p = 0.196) (Fig. 1a). There was no significant difference in the median maternal age of the EPL and CNP samples (10 ± 4.9 and 9.5 ± 6.3 years respectively, p = 0.897), which ranged between 3–12 (EPL) and 2–20 (CNP) years (Fig. 1b). All EPL conceptuses and term placentae came from individual mares, while the CNP conceptuses came from a pool of 7 mares, 3 of whom provided two conceptuses. Male and female conceptuses were equally represented on the array (p = 0.167) (Fig. 1c). DNA samples from the dams of the CNPs were used as presumed diploid adult controls. The five Thoroughbred mares aged 2 to 20 years were reproductively sound.

### Concordance of SNP genotypes

**Figure 2:**
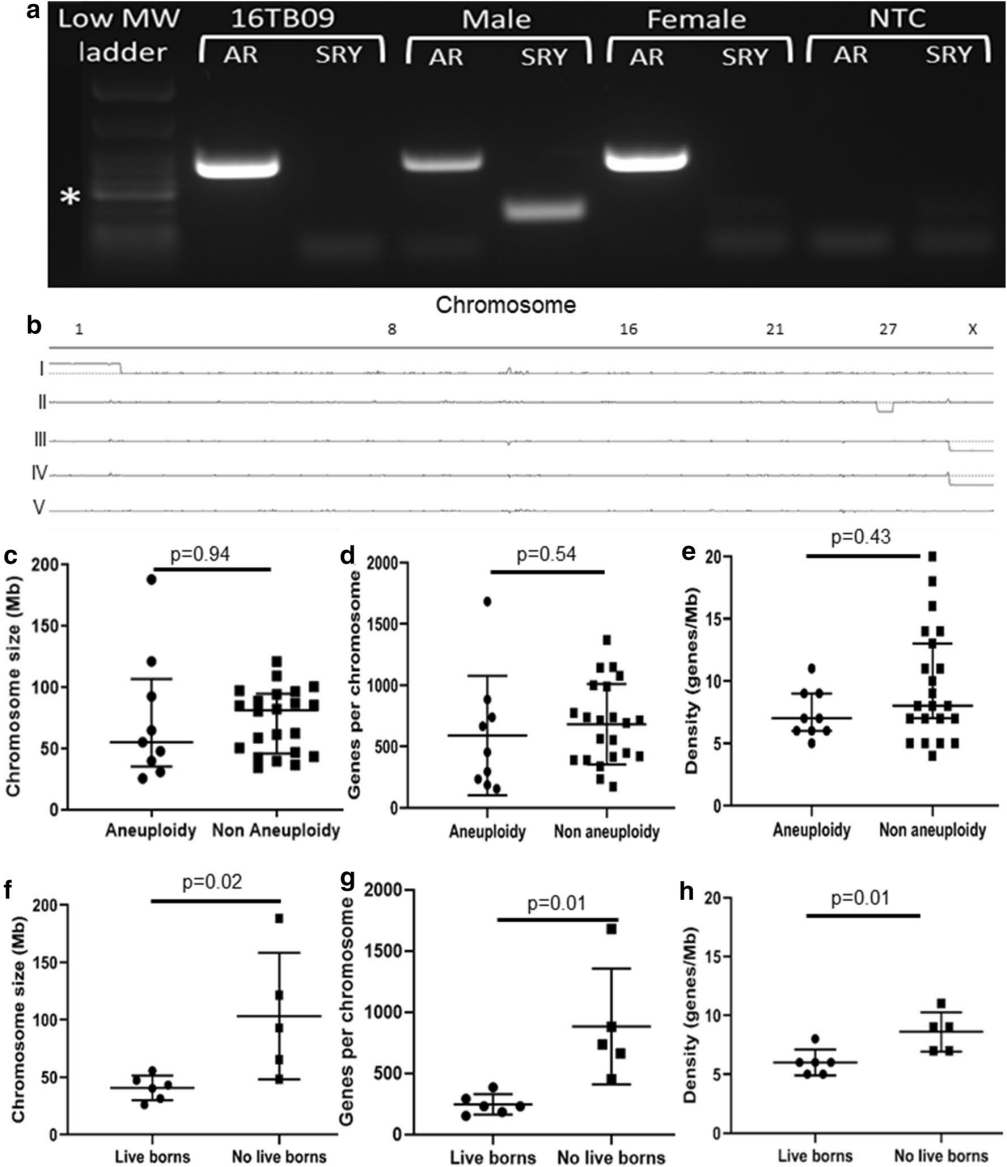
Initial validation of results and identification of aneuploid chromosomes. (**a**) Sex determination using standard PCR with primers for Sex determining Region of Y (*SRY;* Y chromosome; 131 bp) and Androgen Receptor (*AR*; X chromosome; 293 bp) validated the X chromosome copy number status. Confirmed male and female equines as positive controls, and ddH_2_O as no template control (NTC). *200 bp band on low MW ladder. (**b**) Examples of whole genome copy number visualisation with Integrative Genomics Viewer. Chromosome number is displayed horizontally across the top axis, with the centre horizontal line indicating a copy number of 2 (diploid). Allantochorion of (I) female trisomy 1 EPL, (II) female monosomy 27 EPL, and (III) male diploid CNP, along with (IV) male diploid term chorioallantois and (V) female adult peripheral blood mononuclear cells. (**c**–**h**) Analysis of chromosome characteristics comparing (**c**,**f**) chromosome length, (**d**,**g**) the total number of genes, and (**e**,**h**) the gene density per chromosome. Top panel compares the autosomal chromosomes that were found to be aneuploid within the EPL subpopulation of this study to those not identified as involved in aneuploidy (n = 31 for each graph). Bottom panel compares characteristics of aneuploid autosomal chromosomes previously reported in live born equines^21–27^ with those unique EPLs in this study (n = 10 per graph). Mean with standard deviation plotted.

Concordance analysis of the SNP genotype calling was used to assess the accuracy/repeatability of the SNP array (Supplemental Fig. S1). Matching allantochorion (ALC) and fetal samples (from the same conceptus) had a median concordance of 98.7% (range 97.4–99.3) and were significantly different to samples not known to be related (median concordance 70.7%, range 62.9–78.6, p < 0.0001). Samples from pregnancies known to be half siblings were not significantly different from those known to be full siblings (median concordance 74.7 and 81.2, respectively, range 67.0–76.7 and 81.1–83.0, respectively). Tissue repeats (different regions of the same allantochorion) had a median concordance of 99.1% (range 98.9–99.4) while technical replicates (different aliquots of the same DNA sample) had a median concordance of 99.1% (range 99.0–99.4). The sex of the conceptus was determined by PCR (Fig. 2a) and compared with the predicted sex based on the copy number of X chromosome as visualized by Integrative Genome Viewer (IGV) (Fig. 2b). There was 100% agreement (75/75 individuals tested) in the sex of the tissue as determined by PCR and IGV visualisation.

### Aneuploidies identified in failed equine pregnancies

Aneuploidy of at least one chromosome was found in more EPL pregnancies (12/55, 21.8%), as compared with manually terminated early CNPs (0/10, 0%), and healthy term pregnancies (0/5, 0%) (p = 0.057). As an assay control, diploid status was confirmed in all of the reproductively sound adult mares (5/5, 100%) (Fig. 2b, Table 1). Aneuploidies were noted on 10/32 chromosomes (1, 3, 15, 20, 23, 24, 27, 30, 31, and X), representing both trisomies (9/12) and monosomies (3/12). Aneuploidies were noted as single events in each pregnancy (only one chromosomal imbalance event), except for chromosomes 23 and 24 which occurred together in a single failed pregnancy (Table 1). Two of the autosomal aneuploidy types (specific chromosome and ploidy status) found in our EPLs have also been reported in live born equines (trisomies 23^26^ and 30^23,24^ with the remaining seven aneuploidy types being either unique to this study in respect to both specific chromosome and ploidy status (trisomies 1, 3, 15, 20, 24), or involving a previously reported chromosome with a different ploidy status (monosomies 27 and 31) (Table 2).

**Table 1:** Aneuploidy noted in 12/55 early pregnancy loss (EPL) equine conceptuses.

Sample ID	Sex	Gestational Age (days)	Maternal age (years)	Maternal Breed	Aneuploidy noted
14AI01^#^	F	14	4	WB	Trisomy 1
18TB10	M	31	6	TB	Trisomy 3
18TB07*	F	28	19	TB	Trisomy 15
14TB02	M	42	19	TB	Trisomy 20
16TB02	F	58	13	TB	Trisomy 20
18TB09*	M	42	3	TB	Trisomy 23 and 24
16TB09*	F	60	10	TB	Monosomy 27
18TB08	F	42	19	TB	Monosomy 27
16TB03	M	32	19	TB	Trisomy 30
18TB05*	F	41	9	TB	Trisomy 30
18TB15	F	29	10	WB	Monosomy 31
17TB02	F	26	13	TB	Trisomy X

All samples from allantochorion (ALC) except ^#^yolk sac of day 14 conceptus. *conceptuses with both ALC and fetal (F) tissue present on the array, *F* female, *M* male, *TB* Thoroughbred, *WB* Warmblood.

**Table 2:** Comparison of autosomal aneuploidy types.

Phenotype (n)	Aneuploidy type	Chromosome size (Mb)	Genes per chromosome	Maternal age (years)
EPL (1)	Trisomy 1	188.3	1683	4^a^
EPL (1)	Trisomy 3	121.4	883	6^a^
EPL (1)	Trisomy 15	92.9	664	19^a^
EPL (2)	Trisomy 20	65.3	738	13^a^ and 19^a^
EPL (1*)	Trisomy 24	48.4	453	3^a^
EPL (1)	Monosomy 27	40.3	232	10^a^ and 19^a^
EPL (2)	Monosomy 31	26	154	10^a^
EPL (1*), LB (1)	Trisomy 23	55.6	294	3^a^, #^b^
LB (1)	Trisomy 26	43.1	232	3^c^
LB (3)	Trisomy 27	40.3	232	5^d^, 26^e^, #^f^
LB (1)	Trisomy 28	47.3	388	14^g^
EPL (2), LB (2)	Trisomy 30	31.4	185	9^a^, 19^a^, and 23^c^
LB (1)	Trisomy 31	26	154	26^h^

Those unique to our study were larger and contained more genes than those also reported in live born equines. Mares of a variety of ages were affected with an aneuploidy pregnancy. *EPL* early pregnancy loss, *LB* live born, *same conceptus trisomic for two different chromosomes, ^#^unreported mare age.

^a^This study, ^b ^^26^^,^^c ^^24^^,^^d ^^22^^,^^e ^^21^^,^^f ^^23^^,^^g ^^27^^,^^h ^^25^.

Aneuploidy was detected in both large and small autosomes (range 26–188 Mb), along with chromosome X. There was no significant difference between the mean length (± SD) of the autosomal chromosomes detected as aneuploidy compared with those undetected (74.4 ± 52.4 and 73.3 ± 26.0 Mb respectively, p = 0.936) (Fig. 2c). The mean number of genes per autosomal chromosome did not differ significantly between the aneuploidy and non-aneuploidy chromosomes (587 ± 487 and 682 ± 327 respectively, p = 0.536) (Fig. 2d). The median density of autosomal chromosomes did not differ significantly between those detected as aneuploidy and those not detected (7 ± 2.0 and 8 ± 4.5 genes per Mb, respectively, p = 0.433) (Fig. 2e).

The mean length (± SD) of autosomal chromosomes previously reported in aneuploidy live born foals^21–27^ (40.6 Mb ± 10.71) were significantly shorter than those that are unique to this study and only reported in EPL conceptuses (103 Mb ± 55.0) (p = 0.022) (Fig. 2f). The mean number of genes on each autosomal chromosome was significantly different between chromosomes reported in live born^21–27^ and those unique to this study (248 ± 83.7 and 884 ± 473 genes per chromosome, respectively, p = 0.010) (Fig. 2g). Autosomal chromosomes not previously documented in aneuploidy individuals had on average a significantly higher gene density compared with those that have also been reported in the literature^21–27^ (6 ± 1.1 and 8.6 ± 1.7 genes per Mb, respectively, p = 0.013) (Fig. 2h).

### Validation of SNP array results by WGS and ddPCR

**Figure 3:**
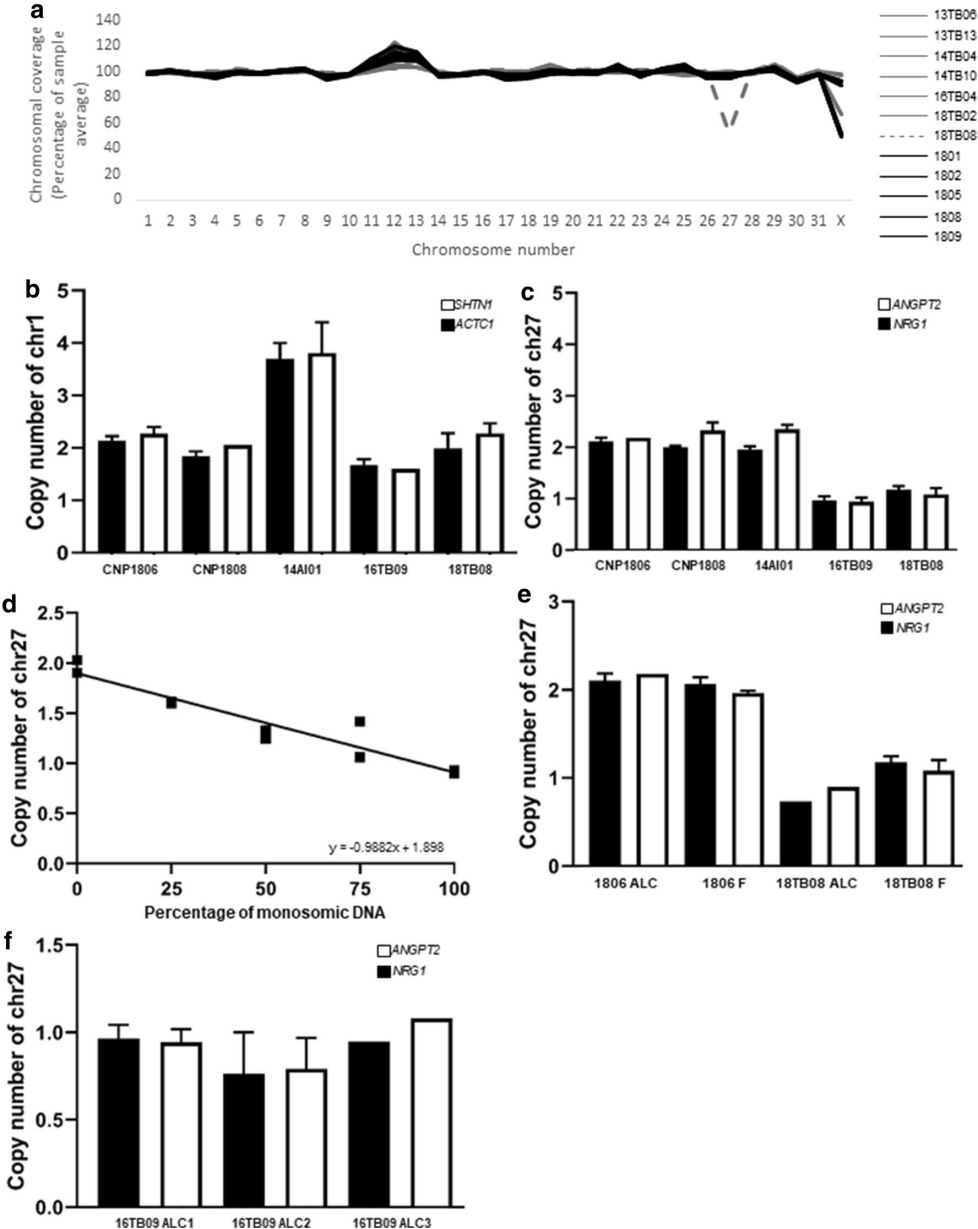
Validation of results by Whole Genome Sequencing and digital droplet PCR. (**a**) Whole genome sequencing (WGS) of 12 of the array samples (n = 1 aneuploidy EPL grey dashed line, n = 6 non aneuploidy EPL grey, n = 5 CNP black). Average coverage per chromosome calculated with SAMtools^65^. Graph presents the chromosome as a percentage of the autosomal average. Digital droplet PCR (ddPCR) of (**b**) chromosome 1 genes *ACTC1* and *SHTN1*, and (**c**) chromosome 27 genes *NRG1* and *ANGPT2* across 5 samples (n = 2 CNP, n = 1 trisomy 1 EPL, n = 2 monosomy 27) relative to the reference region (*MCM6*) on chromosome 18. Primers were designed for two regions at the end of each chromosome. All samples were analysed in duplicate. (**d**) ddPCR of chromosome 27 (*NRG1*) in duplicate. Diploid and monosomy 27 DNA was mixed at different ratios to represent varying levels of mosaicism. Copy number was normalised to the *MCM6* reference region on chromosome 18 and all samples were analysed in duplicate. Negative correlation was noted between the copy number of chromosome 27 and increasing concentration of monosomic DNA (R =  − 0.9882, p < 0.0001). (**e**) DNA from allantochorion (ALC) and fetus (F) of two different conceptuses (n = 1 diploid CNP, n = 1 monosomy 27 EPL), analysed in duplicate for the two regions of chromosome 27 genes. (**f**) DNA from three different regions of allantochorion (ALC) of 16TB09 (monosomy 27) analysed with ddPCR to identify whether conceptus 16TB09 was a mosaic. All regions were analysed in duplicate with chromosome 27 genes and normalised to the *MCM6* reference on chromosome 18. Error bars indicate standard deviation.

Whole genome sequencing (WGS) was performed on five CNP, six EPL diploid conceptuses, and one EPL (18TB08) with monosomy 27 according to SNP genotyping. Average read coverage for chromosome 27 in 18TB08 was approximately half of that for other chromosomes, indicating a monosomy (Fig. 3a). For all other WGS samples without aneuploidy indicated by SNP array analysis (n = 11), the average read coverage for each chromosome was equal (Fig. 3a). Next, digital droplet polymerase chain reaction (ddPCR) was performed to further validate the presence of trisomy 1 (14AI01) (Fig. 3b) and monosomy 27 (16TB09 and 18TB08) (Fig. 3c) using genes on chromosome 18 as a reference. Two control samples (CNP; 1806, 1808) had an approximate copy number of 2 for both genes on chromosome 1 (*ACTC1* and *SHTN1*). The trisomy 1 EPL had an approximate copy number of 3.5 for both genes on chromosome 1 while the two monosomy 27 EPLs that were diploid for chromosome 1 had an approximate copy number of 2. The CNPs (1806, 1808) and the trisomy 1 EPL had an approximate copy number of 2 for both chromosome 27 genes *NRG1* and *ANGPT2* while the two monosomy 27 EPLs had an approximate copy number of 1 for both genes (Fig. 3c).

### Mosaicism is not a common feature of equine aneuploid conceptuses

Aneuploid humans and horses have been shown in some cases to be mosaic^37,38^ so we investigated the ploidy status across fetal tissues. DNA sequences of matching allantochorion (ALC) and fetal (F) tissues from 14 pregnancies (n = 8 EPL, n = 6 CNP) were assessed using IGV. Where aneuploidies were identified in the ALC gDNA (16TB09, 18TB05, 18TB07, 18TB09) the same aneuploidies were always noted in the fetal gDNA. Matching ALC and F tissues of non-aneuploid conceptuses, also shared the same diploid status (n = 10). As a control, an artificial mosaic (varying ratios of normal and aneuploid DNA) was analysed by ddPCR and demonstrated what a mosaic monosomy result would look like when analysed with ddPCR. The aliquot with 100% diploid DNA had a copy number of 2 for chromosome 27, while the aliquot with 100% monosomic DNA had a copy number of 1 for chromosome 27. The copy number of chromosome 27 was negatively correlated (R =  − 0.9882, p < 0.0001) with the increasing volume of monosomic DNA (Fig. 3d). Digital droplet PCR (ddPCR) for two genes on chromosome 27 (*NRG1* and *ANGPT2*) further confirmed the diploid status of both ALC and F from a CNP conceptus (1808), and the monosomy 27 status of both ALC and F from an EPL (18TB08) (Fig. 3e). Next, we investigated the presence of monosomy 27 in three independent ALC tissue samples collected from different regions of the placentae. All three regions of the ALC tested were found to have monosomy 27 (Fig. 3f).

### Phenotypes of EPL and clinical analysis

**Figure 4:**
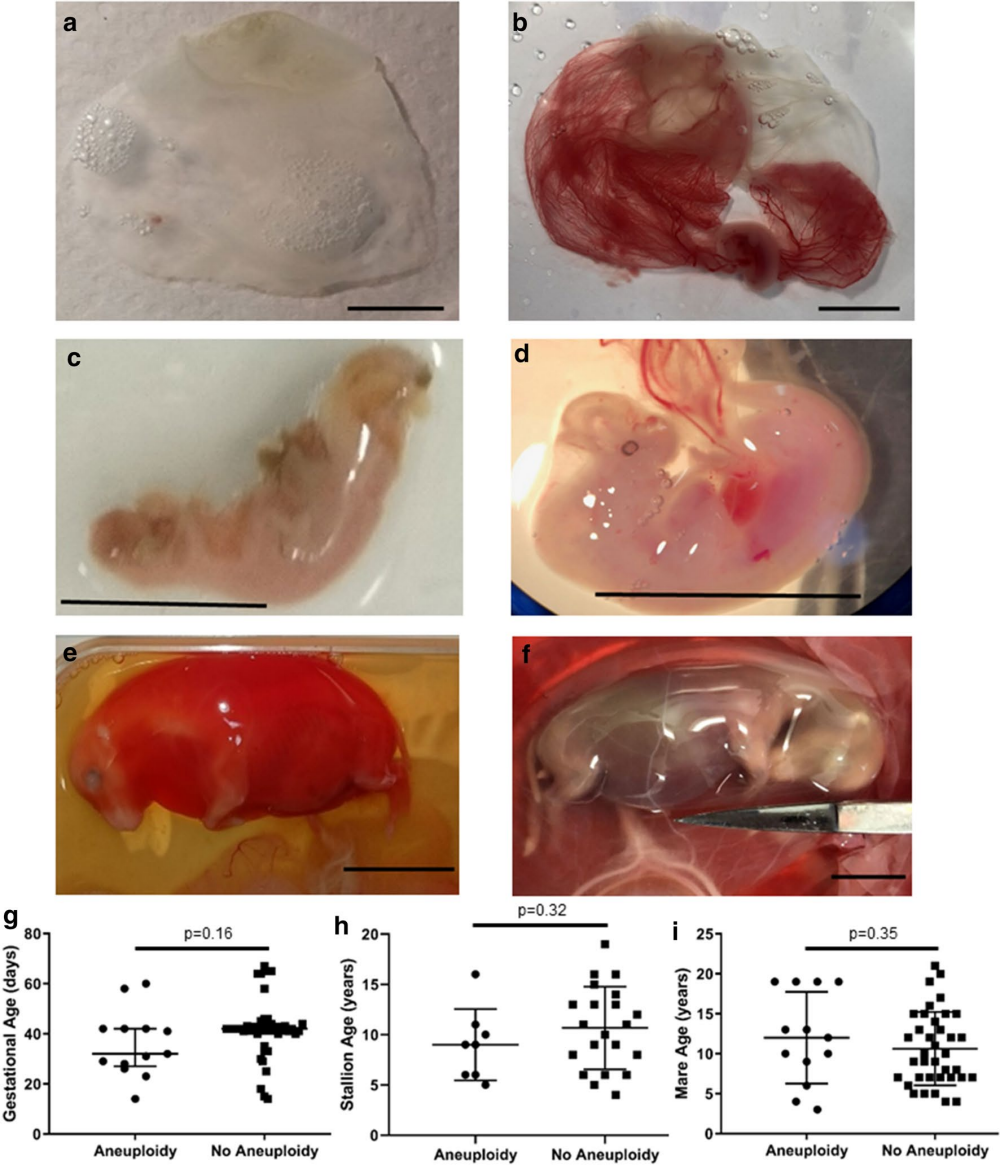
Phenotypes and clinical analysis of aneuploidies. (**a**) Monosomy 31, 29 day gestation failed conceptus and (**b**) age matched 29 day clinically normal conceptus. (**c**) Trisomy 30, 32 day gestation failed embryo proper and (**d**) age matched 33 day clinically normal embryo proper. (**e**) Monosomy 27, 60 day gestation failed fetus and (**f**) age matched 64 day clinically normal fetus. Scale bar = 1 cm for all images. (**g**) Gestational age, (**h**) stallion age, and (**i**) mare age did not significantly differ between aneuploidy EPLs and non-aneuploidy EPLs. Mean with standard deviation plotted.

Of the 55 EPL pregnancies analysed, 34 had a fetus confirmed present within the conceptus. There was no significant difference in aneuploidy occurrence between conceptuses presenting with a fetus and those without a fetus (p = 0.36). Of the 12 aneuploidy conceptuses, three did not have a fetus at dissection; one was too young for an embryo proper (Trisomy 1, 14 days old), one was submitted as a complete and intact conceptus with a suspected embryonic disc only with no evidence of vasculature (Monosomy 31, Fig. 4a—age matched CNP for comparison Fig. 4b), and one had evidence of a fetus recorded in clinical records but no fetus was present at dissection (Trisomy X). One submission was accompanied by autolytic fetal remnants (trisomy 20) and the remaining seven aneuploid EPLs presented with an intact/partially intact fetus of variable phenotypes. A 32 day trisomy 30 embryo proper (Fig. 4c) appeared to have distorted and mismatched developmental features compared with the age matched CNP embryo proper (Fig. 4d), although autolytic changes impaired full assessment of this EPL specimen. The day 60 monosomy 27 fetus (Fig. 4e) appeared oedematous and congested when compared with the day 64 CNP fetus (Fig. 4f) consistent with an abnormal vasculature phenotype.

None of the predictive variables analysed (conceptus sex, maternal age, paternal age, gestational age, breed, use of ovulation induction, twin pregnancies, positive bacterial growth from a uterine swab at time of loss) were significantly associated with risk of aneuploid EPLs (p ranged from 0.34 to 0.57; Fig. 4g–i, Supplementary Table S2). Next, we compared the likelihood of a mare ending the season with a live foal after suffering either an aneuploid EPL or a non-aneuploid EPL. Mares who suffered an EPL after 43 days when endometrial cups are established were excluded. Mares who suffered aneuploidy EPL were significantly more likely to end the season with a live birth (3/4 mares) compared to those with a diploid EPL (2/15 mares) (p = 0.04).

## Discussion

An abundance of reports into human miscarriages cite aneuploidy as the greatest cause of the pregnancy failure^17,39^. In contrast, reports of aneuploidy associated with pregnancy loss in domesticated animals is rarely reported. We identified autosomal aneuploidies in 20% of naturally occurring equine EPLs, lower than reported in women^39,40^ possibly explained by the shorter gestational time frame used in our study. Five trisomies (trisomies 1, 3, 15, 20, and 24) and two monosomies (monosomies 27, and 31) were unique to the study. None of the pregnancies assessed across both fetal and placental compartments were mosaic, collectively providing evidence that these aneuploidies are likely to be embryonic/fetal lethal and zygotic in origin. Additionally, the Axiom Equine 670K SNP Genotyping Array proved to be a successful methodology to identify aneuploidies.

In mammalian species studied to date, very few autosomal aneuploidies are tolerated to term. Whereas partial monosomies have been identified in humans^41^, no cases of complete autosomal monosomy compatible with birth have been recorded in any species. Therefore, it is highly likely the monosomic conceptuses described here involving chromosomes 27 and 31 failed due to decreased gene dosage resulting in embryonic/fetal lethality. While trisomies can present in live borns, the phenotypes vary considerably^42^. In the horse, only eight individual case reports of autosomal aneuploid live borns exist, involving chromosomes 23, 26, 27, 28, 30, and 31. These presented with variable congenital abnormalities involving the musculoskeletal, neurological, and vasculature systems^21–27^. Of these aneuploidy types tolerated to term, only trisomy 30 was also identified in EPL conceptuses. How equine trisomy 30 can cause both fetal lethality and live born phenotype is not known, although studies of phenotypic variation in Down’s Syndrome suggest the elevated transcript levels generated by the genotype could be modified by the environment to initiate and exacerbate the resultant phenotype^43^. The chromosomes with aneuploidy type unique to failed pregnancies were found to be on average significantly larger, more dense, and with more genes compared with those that present a mixed phenotype (EPL/live born). This is consistent with the hypothesis that duplication or deletion of larger chromosomes results in greater genetic imbalances and hence earlier lethality. Indeed, amongst our autosomal trisomies, those involving the largest chromosomes (trisomy 1, 3, and 15) were from pregnancies that failed at the youngest gestational ages (days 14, 32, and 28, respectively). The equine pregnancies assessed here were all clinically recognised, therefore incidence of aneuploidy in equine pregnancy is predicted to be higher than the 22% we report. In support of this, a report identified aneuploidy of larger chromosomes (2 and 4) in in vitro and in vivo generated equine blastocysts^30^. These aneuploid types were not identified here but given the chromosome sizes, it is plausible that their phenotype is embryonic lethal prior to clinical detection.

The combination of aneuploidy type, the degree of mosaicism, and the tissue compartment location determines the severity of the phenotype^44^. Mosaicism, and the percentage of the cells that it inhabits, offers an insight into the initial starting point of the aneuploidy, with a negative correlation between developmental time at error and the degree of mosaicism. For a true aneuploidy (100% of cells containing the imbalance), the imbalance must have occurred prior to conception. The imbalance more commonly occurs within the oocyte (during meiosis I or II), with as high as 93% of human trisomy 21 cases being maternal in origin, but can also occur within spermatocytes^45^. As mosaic autosomal aneuploidies have been reported in adult equines^46^, we tested the matching allantochorion and fetus of eight EPL and six CNP conceptuses, and multiple allantochorion samples from two separate conceptuses using a combination of SNP analysis and ddPCR. The aneuploidy status was consistent between and within tissue compartments in all individual pregnancies tested, indicating a true aneuploidy instead of a mosaic and suggestive of maternal or paternal origin, as is commonly found in humans^45^.

Advancing maternal age is associated with decreased quality of oocytes^47^, from altered epigenetic profiles^48^ and mitochondrial DNA deletions^49^. Aged oocytes also display altered microtubule spindle alignment^50^ and weakened centromere cohesion^51^ resulting in increased risk of aneuploidy. In the mare, advancing maternal age increases the risk of chromosome misalignment^16^ and early pregnancy loss^5^. Whilst numerically there was an increase in the proportion of aneuploid pregnancies in older mares, there was also an increase in the proportion of very young mares with aneuploid pregnancies and neither were statistically different to the non-aneuploid EPL pregnancies. This may reflect non-aneuploid EPLs being at increased risk with advanced maternal age, as age is associated with increased endometrial disease^52^. The identification of embryonic lethal aneuploidy EPLs in very young mothers has been reported in human medicine^53^. While advancing paternal age has been demonstrated to increase allosome aneuploidy rates in equine spermatozoa^54^, whether this leads to sex chromosome aneuploidy in the offspring or pregnancy losses is not currently known. Similarly to a study in human miscarriage^55^,we found no association with an increased risk for equine autosome aneuploidy in EPLs, however paternal age may still influence the risk of smaller genetic abnormalities.

Of the 20 individuals with autosomal aneuploidy reported to date (combining reports in the literature with our study), approximately 45% involve chromosome 27 or 30 (five trisomy live born^21–24^, two monosomy 27 EPLs, and two trisomy 30 EPLs). The underlying mechanisms of chromosomal susceptibility to aneuploidy is not yet known however, chromosomes 27 and 30 are among the most gene poor equine autosomes. Phenotypic differences are interesting as trisomies of chromosome 27 can be viable to term, displaying varying characteristics within 24 months of birth including cryptorchidism, inability to suckle, skeletal abnormalities, atypical gait, and reduced social skills^21–23^. Our study added to the list of chromosome 27 aneuploidies by documenting two monosomies in EPLs, both displaying extreme oedema with intact vasculature. All four of the trisomy 30 individuals display skeletal abnormalities, suggesting the genetic imbalance associated with trisomy 30 disrupts mechanisms involved with correct skeletal development^23,24^. The phenotype of the identified aneuploid EPLs was variable with gestational ages (ranging from 14 to 64 days), fetal and clinical presentations. Contrary to our initial hypothesis, aneuploidy EPLs were not significantly associated with an anembryonic status, with only 1/12 aneuploidy pregnancies phenotypically presenting as anembryonic. Based on the variables we studied, an aneuploidy pregnancy is clinically indistinguishable from diploid pregnancies. Unfortunately, due to low numbers, we were unable to investigate clinical features associated with a particular aneuploidy subtype. We found that mares who suffered an aneuploid EPL were significantly more likely to carry the next pregnancy to term, compared to those who lost a diploid pregnancy. This suggests that aneuploidy events occur sporadically in some mares. This finding is similar to observations in women, who have a small increased chance of having a live birth in the subsequent pregnancy following aneuploidy miscarriage^56^.

While mice models of oocyte ageing has led to evidence of DNA fragmentation^57^ and altered gene expression patterns^58^, the fundamental differences between rodents and humans may limit transfer to human medicine. Rodents are polytocous and display differing endocrine profiles compared with humans. Mares, on the other hand display similar endocrine profiles (notably follicle stimulating hormone levels) to women, particularly with regards to ageing^59^. As discussed above, advanced maternal age is associated with higher incidence of pregnancy loss in both species^2,13^. Our study highlights that aneuploidy rates are similar between human and horse natural occurring pregnancy losses, show a similar propensity to be maternally or paternally derived, and a possible bias towards very young and old mothers being at risk.

In conclusion, our results provide the first evidence of true aneuploidy in naturally occurring equine pregnancy losses. We suggest the horse as a viable alternative to study underlying mechanisms of aneuploidy and to test future novel therapeutics, for application in human medicine.

## Materials and methods

### Sample acquisition, handling and phenotyping

Conceptus material from 55 early pregnancy losses (EPLs) were submitted from veterinarians across the UK and Ireland (2013–2018 breeding seasons). Pregnancies, confirmed by transrectal ultrasound at 14–16 days post ovulation, and subsequently lost before 65 days post ovulation were included in this study. Following confirmation of pregnancy failure (no heartbeat/collapsed vesicle), conceptuses were recovered by non-invasive uterine lavage by the attending veterinarian according to previously established protocols^35^ before being transported to the Royal Veterinary College (RVC) in sterile transport media medium (Hank’s Balanced Salt Solution, 5% FBS, Amphotericin B 250 µg/ml, 10,000 units/ml Penicillin, 10,000 µg/ml Streptomycin, Kanamycin). Reproductive histories of the mares were obtained from the submitting veterinary practices and stud farms.

Control pregnancies comprised of manually terminated clinically normal pregnancies (CNP) and term placentae following the delivery of a healthy foal (term). Two CNPs came from pregnant Thoroughbred broodmares euthanised for complications unrelated to pregnancy. Five Thoroughbred mares (aged 2–20 years) housed at the Biological Services Unit of the RVC (kept on grass *ad lib*, supplemented with hay over the winter) were donors for the remaining eight CNPs (2018 breeding season). Each mare tested negative for Equine Viral Arteritis (EVA)/Equine Infectious Anaemia (EIA), *Taylorella equigenitalis*, *Pseudomonas aeruginosa*, and *Klebsiella pneumonia* (Rossdales Laboratories, Newmarket, UK) prior to the onset of the stud season. Ovulation was induced with 1,500 IU of human chorion gonadotropin (hCG, Chorulon) administered intravenously and mares were artificially inseminated 24 h later with a commercial dose of chilled semen from Thoroughbred stallions of proven fertility. Transrectal ultrasound confirmed pregnancy 14 days post ovulation and conceptus development was followed by subsequent ultrasound examinations twice weekly. Clinically normal developing pregnancies (presence of corpus luteum, absence of intrauterine fluid, appropriately sized embryonic vesicle, detection of embryo proper, and appropriately timed detection of fetal heartbeat) were manually terminated between 29 and 41 days gestation and recovered, as previously described^60^. EPL and CNP conceptuses were washed three times in PBS (containing 10 units/ml Penicillin and 10 µg/ml Streptomycin) before the tissues were identified and dissected. Tissues harvested depended on the developmental age and completeness of the submitted conceptus. When available, they included chorion, allantochorion, yolk sac, chorionic girdle, and fetus. Placenta from five healthy term births (2017 stud season) were collected from stud farms in Hertfordshire and Suffolk and brought to the RVC for dissection. Sections were washed three times in PBS and then snap frozen in approx. 5 × 5 mm sections in liquid nitrogen before storage at − 80 °C.

Whole blood from five mares (dams of CNP conceptuses) presumed to be diploid due to having normal fertility and being in good general health was collected in heparin vials and peripheral blood mononuclear cells (PBMCs) were isolated as previously described^61^. PBMCs were then snap frozen in liquid nitrogen and transferred to − 80 °C for long term storage.

### DNA extraction, and sexing of conceptuses

DNA from placenta (allantochorion and chorion), fetus, and PBMCs were extracted using QIAGEN DNeasy Blood and Tissue kit (Qiagen Sciences, Maryland, USA), following manufacturer’s guidelines. Briefly, tissue or cells were incubated at 56 °C overnight in buffer ATL and proteinase K. Incubation at room temperature for 2 min with 28 U RNase A as recommended by the manufacturer proceeded passage through a spin column, before elution with 100 µl Buffer AE. DNA was quantified using a DeNovix Spectrophotometer.

Conceptuses were sexed by standard PCR as previously reported^62^. Briefly, primers for the Sex determining Region of Y (*SRY*) were used to determine the presence (male) or absence (female) of *SRY* from 50 ng of total genomic DNA (Supplementary Table S1) using FIREPol DNA polymerase (Solis Biodyne, Estonia). Primers for the X-linked Androgen Receptor *(AR*) gene were used as positive control for DNA quality. DNA from male and female equines served as positive controls, while ddH_2_O was the negative control. PCR conditions were as follows: 95 °C (5 min), 40 cycles of: 95 °C (30 s), 60 °C (40 s), and 72 °C (1 min), followed by 72 °C for 10 min. Amplicons were resolved on a 2% agarose gel and visualised in ultraviolet light.

### HD 670K equine SNP array

The 55 EPL samples genotyped on the Axiom Equine 670 K SNP Genotyping Array^36^ (25 male and 30 female) were from Thoroughbred, Warmblood, and unknown breed pregnancies, with a spread of gestational and mare ages (range 3–21 years). Samples from 10 individual Thoroughbred CNP conceptuses (five male and five female), and five healthy TB term placentae (three male and two female) were genotyped to represent placenta from viable pregnancies. Of the 65 EPL/CNP conceptuses, 14 had matching fetal and placental DNA genotyped by the array. Equal loads of DNA (760 ng) were hybridised along with fluorescent probes (PE at 660 nm and FAM at 578 nmto the array. One conceptus was represented five times to act as both tissue (three individual sections of allantochorion; ALC) and technical (same DNA aliquot represented three times) replicates. PBMCs from five reproductively sound adult Thoroughbred mares (age range 2–20 years) were also genotyped to confirm their diploid status and to provide assay controls.

Raw intensity .CEL files were imported in Axiom Analysis Suite (AxAS; v5.0.1.38, ThermoFisher, UK, https://www.thermofisher.com/uk/en/home/life-science/microarray-analysis/microarray-analysis-instruments-software-services/microarray-analysis-software/axiom-analysis-suite.html). Following the Copy Number Discovery workflow (Supplementary Table S3) which estimates copy number based on the deviation of probe fluorescence intensity for each marker on the array to the average probe intensity, copy number estimations were imported into Integrated Genome Viewer (IGV; v, Broad Institute, https://software.broadinstitute.org/software/igv/) with EquCab3.0 reference genome. Whole genome visualisation allowed for identification of aneuploidies. Quality control metrics were in place using AxAS, with 86.46% pass rate for the samples.

### Whole genome sequencing

Twelve samples (n = 7 EPL, n = 5 CNP) also underwent whole genome sequencing (WGS) using Illumina 150 bp paired-end sequencing on the NovaSeq 6,000 targeting an average genome coverage of 30X . Data were then aligned to EquCab3.0 using SpeedSeq^63^, and read depth was estimated using SAMtools^64^ genome coverage function to determine chromosome ploidy. EPL samples selected for WGS had a phenotype of Thoroughbred, gestation age between 30 and 42 days, and negative uterine swab at loss, with equal representation of conceptus sex and maternal age. CNP conceptuses were age, sex, and breed matched to EPL samples.

### Digital droplet PCR

Digital droplet PCR (ddPCR) was performed using a BioRad QX200 system (BioRad, UK). Primers were designed for two genes per chromosome of interest: *NRG1* and *ANGPT2* (chromosome 27), *SHTN1* and *ACTC1* (chromosome 1), with *MCM6* (chromosome 18) as the reference (all individuals were diploid for this chromosome) (Supplementary Table S1). Copy number was determined in duplicate in reactions containing 50 ng DNA, using EvaGreen chemistry (final concentration: 100 nM each primer, 1 × ddPCR Supermix for EvaGreen). C1000 Touch Thermal Cycler performed ddPCR reactions as follows: 95 °C (5 min), 40 cycles of: 95 °C (30 s) and 58 °C (1 min), followed by 4 °C (5 min), and 90 °C (5 min). Droplets were analysed using Bio-Rad QX200 Droplet Reader and QuantaSoft Analysis Pro (v1.0.596; BioRad, https://www.bio-rad.com/en-uk/product/qx200-droplet-digital-pcr-system?ID=MPOQQE4VY). The copy number of each product was manually calculated relative to *MCM6*.

### Statistical analysis

Normality of the numerical variable was assessed using Shapiro–Wilk normality test. Two sample t-test (or Mann–Whitney U test where normality test failed) was performed to compare groups. Simple linear regression was used for the mosaicism. These analyses were carried out using GraphPad Prism (v7.03, https://www.graphpad.com/). Association between categorical predictors (sex of the conceptus, mare status at the beginning of the season, mare age, breed, use of ovulation induction, twin pregnancy, and uterine infection) and whether the mare suffered aneuploid EPL was evaluated using Fisher’s exact test. The relationship between aneuploidy EPLs and the likelihood of a mare ending the season with a foal was also evaluated using Fisher’s exact test (SPSS v26, IBM, https://www.ibm.com/analytics/spss-statistics-software). Significance was set at p < 0.05 for all analysis. Due to the low numbers, barren (did not produce a live foal from previous season) and rested (deliberately not bred) mares were combined into one group and compared with maiden (never bred) and foaled (produced a live foal from previous season) mares.

### Ethical approval

All conceptus recoveries from clinical cases of pregnancy loss were performed with written informed owner consent under ethics approval from the Clinical Research and Ethical Review Board at the Royal Veterinary College (URN:2012-1169 and URN:2017-1660-3). Animal care and conceptus recoveries from the clinically normal pregnancies were performed in accordance with the Animals (Scientific Procedures) Act 1986 guidelines set by 8 the Home Office and Ethics Committee of the Royal Veterinary College, London (HO licence PPL 70/8577).

## Supplementary information

Supplementary Legends

Supplementary Information

## Data Availability

Raw .CEL files from the five Thoroughbred mares along with their CNPs can be found in this public repository https://doi.org/10.34840/mnah-vv94, along with processed SNP array data from submitted samples (EPLs and term placentae). Whole genome sequences from the five CNPs can also be found at the same repository, along with coverage data for the remaining WGS samples. Raw ddPCR data may also be found here. Raw data from the submitted samples cannot be shared publically as they were provided under an owner consent agreement that guaranteed anonymity due to the commercial sensitivity of the data. Partial sequences/genotypes that can be released without compromising confidentiality will be made available through the corresponding author, Dr Amanda de Mestre, The Royal Veterinary College (ademestre@rvc.ac.uk).
