# Supplementary material for: Whole genome analysis reveals aneuploidies in early pregnancy loss in the horse
Source: Sci Rep. 2020 Aug 7;10:13314. doi: 10.1038/s41598-020-69967-z (PMC7415156; doi:10.1038/s41598-020-69967-z)
Supplement: Supplementary file 1 — Supplementary Legends [file 41598_2020_69967_MOESM1_ESM.docx]

**Supplementary Table 1. Primer details.** Details of primers used for both standard polymerase chain reaction (PCR) and digital droplet PCR (ddPCR).

**Supplementary Table 2. Analysis of clinical variables**. Univariable analysis of nine clinically relevant variables using Fisher’s Exact revealed no significant variable that could predict aneuploidy. Mares who suffered aneuploidy EPL were significantly more likely to end the season with a live birth (3/4 mares) compared to those with a diploid EPL (2/15 mares) (p=0.04).

**Supplementary Table 3. Copy Number Discovery workflow argument values**. Argument values used in Axiom Analysis Suite (AxAS; ThermoFisher, UK) to estimate copy number based on the deviation of probe fluorescence intensity for each marker on the array to the average probe intensity.

**Supplementary Figure 1. Concordance analysis of the SNP calls for each sample**. Samples from the same conceptus (“Match” – matching fetal and allantochorion DNA) were significantly different to samples that were not known to be related at all (“Unrelated”, p<0.0001). “Half-sib” compared the concordance of individuals sharing one parent, while “Full-sib” compared those that shared both parents. “Bio rep” indicates the comparison of three regions across the allantochorion of a single conceptus, while “Tech rep” compared a single aliquot of DNA from the same region of allantochorion of a single individual. “Mat-Con” compared the concordance of maternal DNA with the corresponding offspring DNA. Median with interquartile range plotted.
