# Supplementary material for: Whole genome analysis reveals aneuploidies in early pregnancy loss in the horse
Source: Sci Rep. 2020 Aug 7;10:13314. doi: 10.1038/s41598-020-69967-z (PMC7415156; doi:10.1038/s41598-020-69967-z)

Whole genome analysis reveals aneuploidies in early pregnancy loss in the horse

Charlotte A. Shilton[1], Anne Kahler[1¶], Brian W. Davis[2¶], James R. Crabtree[3], James Crowhurst[4], Andrew J. McGladdery[5], D. Claire Wathes[6], Terje Raudsepp[2], Amanda M. de Mestre[1*]

1. Department of Comparative Biomedical Sciences, The Royal Veterinary College, University of London, United Kingdom
2. Department of Veterinary Integrative Biosciences, Texas A&M University, College Station, Texas, United States of America
3. Equine Reproductive Services (UK) Ltd., North Yorkshire, United Kingdom
4. Newmarket Equine Hospital, Newmarket, Suffolk, United Kingdom
5. Rossdales Equine Practice, Newmarket, United Kingdom
6. Department of Production and Population Health, The Royal Veterinary College, University of London, United Kingdom

¶Authors contributed equally to the work
*Corresponding author
E-mail: ademestre@rvc.ac.uk

Supplementary Table 1

Assay	Target	Chromosome	Product Size (bp)	Annealing temperature (°C)	Primer Sequence
Standard PCR	AR	X	293	60	F: AGCAGCAACAGGAGACCAG<br>R: TGCTTAAGCCTGGGAAAGTG
Standard PCR	SRY	Y	131	60	F: TGCATTCATGGTGTGGTCTC<br>R: ATGGCAATTTTTCGGCTTC
ddPCR	ACTC1	1	146	58	F: CAAGCAGGAGTACGATGAGG<br>R: TGGATGAGAGATGAGGGAGG
ddPCR	SHTN1	1	113	58	F: ATCTCAGAGTTGACCAGGGT<br>R: TGGCTGTAACAGTCACTTGG
ddPCR	MCM6	18	120	58	F: ACTTCCATAGGAGCCTCCAA<br>R: AAACGTGAGGGAAGAACTGG
ddPCR	NRG1	27	136	58	F: CTGTGACTTAGCGTCTGGAG<br>R: TTATGGTCTTGGGAGGTCCA
ddPCR	ANGPT2	27	116	58	F: CCATGATAGTTAGCGCACCA<br>R: AGTGTGTTTACTGTCACCGG

Supplementary Table 2

Variable	Categories	Sample size			Total n	p value	Significant
		Aneu	Non aneu	Group			
Sex of conceptus	Male	4	21	25	55	0.514	N
	Female	8	22	30			
Maternal Age (3 cat)	Young	2	3	5	51	0.426	N
	Middle age	6	27	33			
	Old	4	9	13			
Maternal Age (2 cat)	Young & old	6	12	18	51	0.338	N
	Middle age	6	27	33			
Status	Maiden	2	7	9	46	0.558	N
	Barren/rested	3	16	19			
	Foaled	6	12	18			
Foal at start of season	Yes	3	16	19	46	0.457	N
	No	8	19	27			
Ending season with live foal	Yes	3	2	5	19	0.0374	Y
	No	1	13	14			
Ovulation induction	Yes	9	23	32	41	0.477	N
	No	1	8	9			
Twins	Yes	1	3	4	42	0.404	N
	No	10	28	38			
Growth from uterine swab	Yes	2	3	5	33	0.57	N
	No	6	22	28			
Fetus at dissection	Yes	8	27	35	48	0.361	N
	No	4	9	13			

Supplementary Table 3

Argument	Value
CN Reference File	Axiom_MNEc670.r3
CN Regions File	Axiom_MNEc670.r3.hmm_regions
MAPD	≤0.35
Waviness SD	≤0.1
seg-min-bases-CN-oneormore	≥50000
seg-min-bases-CN-zero	≥25000
seg-min-probesets-CN-oneormore	≥50
seg-min-probesets-CN-zero	≥25

Supplementary Figure 1

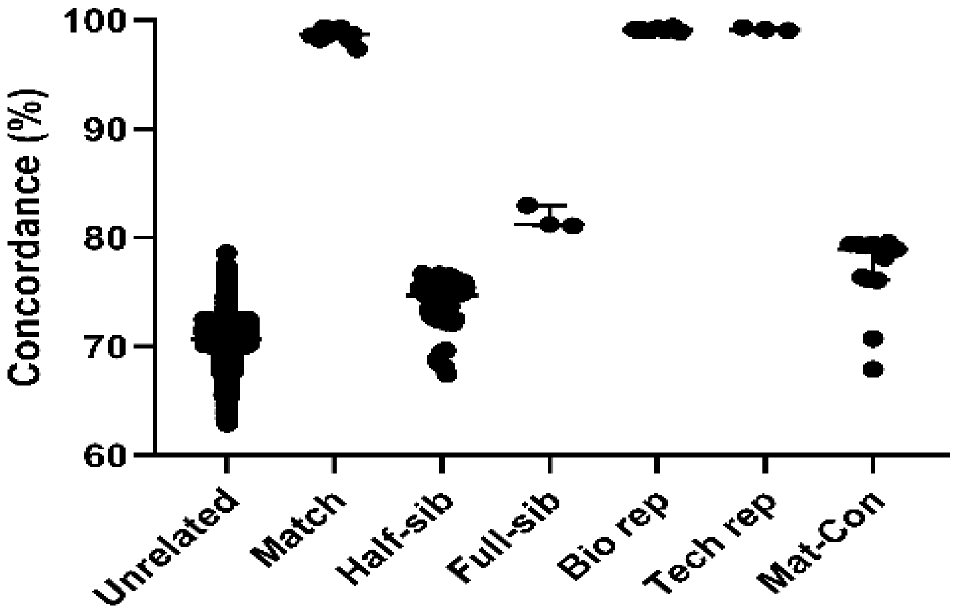

Supplementary uncropped gel (figure 2a)

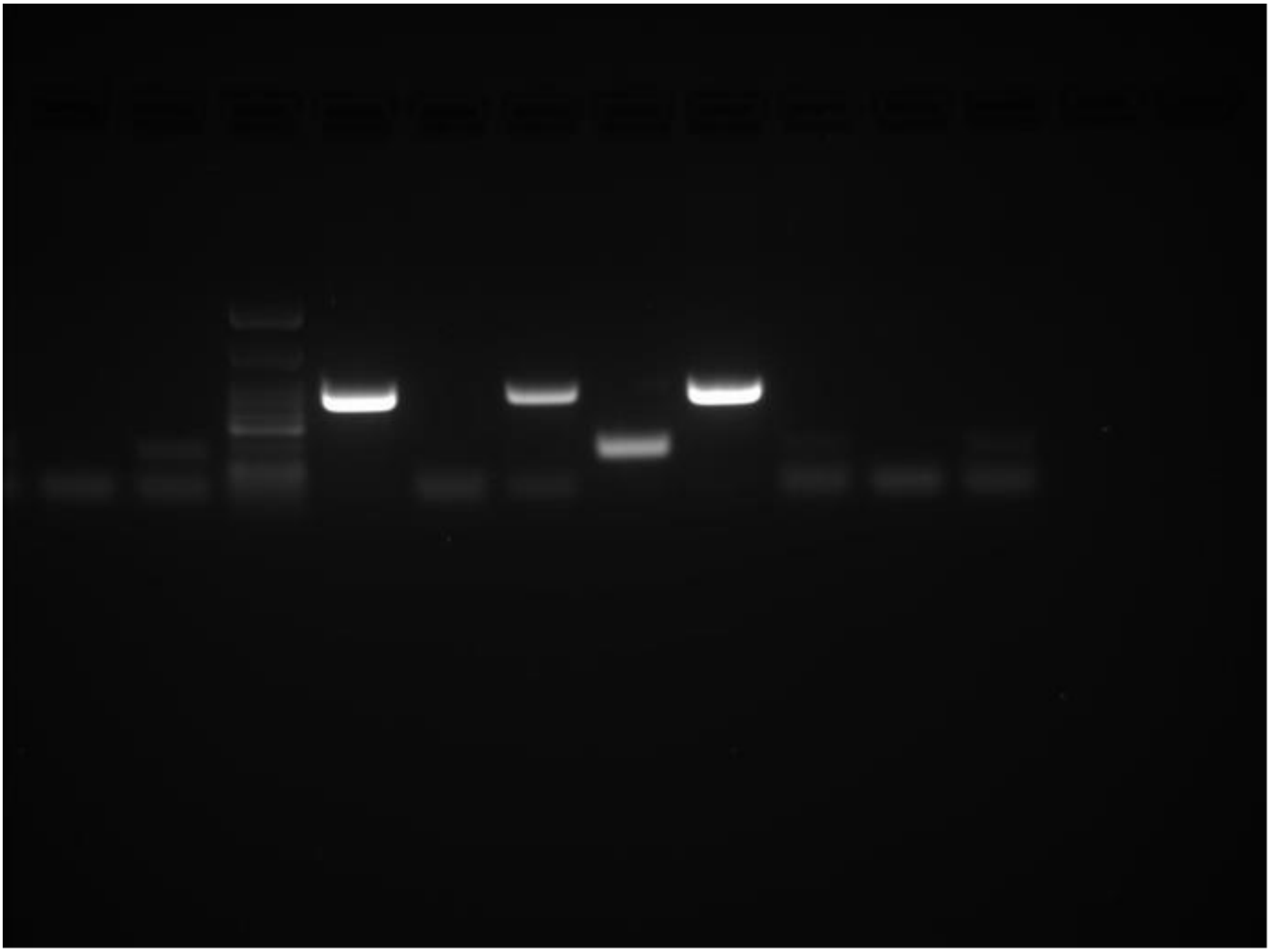

Supplement: Supplementary file 2 — Supplementary Information [file 41598_2020_69967_MOESM2_ESM.pdf]
